# Does targeted information impact consumers’ preferences for value-based health insurance? Evidence from a survey experiment

**DOI:** 10.1186/s13561-024-00573-9

**Published:** 2024-11-18

**Authors:** Tess L. C. Bardy, Stefan Boes

**Affiliations:** Faculty of Health Sciences and Medicine & Center for Health, Policy, and Economics, University of Lucerne, Alpenquai 4, Lucerne, CH-6005 Switzerland

**Keywords:** Information provision, Health insurance preferences, Value-based insurance design, Choice-based health insurance, Health insurance literacy

## Abstract

**Objectives:**

Value-based insurance design (VBID) aims to direct consumers’ preferences by incentivizing the use of high-value care and discouraging the use of low-value care. However, consumers often have limited knowledge of health insurance and the health insurance system, possibly distorting their preferences. In this study, we aim to investigate the impact of specific information treatments on consumers’ preferences for VBID.

**Methods:**

We implemented an information experiment as part of a representative survey on health insurance literacy and preferences for VBID within Switzerland’s choice-based health insurance system. Preferences for VBID were measured through a discrete choice experiment. Cross-sectional data on 6,033 respondents aged 26–75 were analyzed using descriptive statistics and mixed logit regressions.

**Results:**

Respondents showed strong preferences for their current health insurance instead of VBID alternatives. A general description of current regulations on cost-sharing, drug disbursement, and monthly premiums significantly increased preferences for VBID (*p* < 0.01). Pointing respondents specifically to VBID further reduced the opposition against VBID plans. At the same time, there is evidence for anchoring effects in copayments after receiving the information treatments, irrespective of the value of the care.

**Conclusions:**

The results of this study highlight that individuals are susceptible to provided information about health insurance when building their preferences for VBID. One potential explanation is limited health insurance literacy, implying that tailored communication strategies may be needed to improve insurance decision-making.

**JEL Classification:**

I11, I13.

**Supplementary Information:**

The online version contains supplementary material available at 10.1186/s13561-024-00573-9.

## Highlights

People generally prefer their current health insurance plan over alternative plans that incorporate value-based features, such as incentivizing the use of high-value care.

Providing detailed information about these new types of health insurance plans shifts people’s preferences toward them, suggesting a need for more comprehensive knowledge about health insurance.

The observed preference shifts underline the importance of improved communication strategies to facilitate informed decision-making in health insurance.

**Supplementary Information:**

The online version contains supplementary material available at 10.1186/s13561-024-00573-9.

## Introduction

Health insurance literacy (HIL) is crucial in ensuring informed decisions and efficient utilization of health insurance plans within choice-based health insurance systems. HIL refers to individuals’ ability to seek, understand, evaluate, and effectively use health insurance-related information [1]. Limited HIL can create significant barriers to selecting and using health insurance plans, resulting in financial burdens and an increased likelihood of delaying, if not forgoing, necessary care [2–4].

To address limited HIL and guide consumers in insurance decision-making, the integration of value-based incentives into health insurance plans, such as value-based insurance design (VBID), has been suggested [5, 6]. For example, VBID may employ cost-sharing options to direct consumers’ preferences for certain healthcare services, medications, and physicians [7]. This approach recognizes the varying clinical benefits associated with different medical services, aiming to incentivize the utilization of high-value care (through lower cost-sharing) and discouraging low-value care (through higher cost-sharing), thus improving health outcomes, avoiding the provision of wasteful or even harmful care, and controlling healthcare costs [8, 9].

Despite the potential benefits of VBID [10], its successful implementation depends on understanding consumers’ preferences for VBID. It has been shown that information provision plays a critical role in shaping preferences and influencing decision-making across various domains [11–13], including health insurance [14, 15]. Providing health insurance-related information reduces information frictions and improves health insurance navigation, allowing individuals to make better decisions according to their health and financial needs [12, 13, 15–17].

However, research examining the impact of targeted information provision on individuals’ preferences in the context of health insurance remains limited [14]. Our study aims to address this gap by investigating individuals’ preferences for VBID and whether they change if additional information about the insurance options is provided. If individuals had a good understanding of insurance concepts and terms and the health insurance system, providing them with additional information should not alter their preferences. However, if individuals’ preferences change when additional information is provided, this indicates limited HIL, which has implications for the design and implementation of VBID. We employ an experimental design where respondents took part in a discrete choice experiment (DCE) to measure their preferences for VBID after being randomly assigned to a control and two information treatment groups. Depending on their group allocation, respondents faced specific information provisions about health insurance and its attributes relevant to the VBID elements in the hypothetical health plans offered [18].

Switzerland’s choice-based health insurance system provides a unique opportunity to develop this experiment. Individuals are obliged to purchase basic health insurance from a regulated selection of plans defined by the Swiss Federal Law on Compulsory Health Care. Within a managed competition market structure, insurance premiums are community-rated. They can vary across insurers, primarily based on cost-sharing and plan type (free choice of provider or managed care), and by three age groups (children, adolescents, adults) within a premium region. Cost-sharing involves a CHF 300 yearly basic deductible and five optional higher deductible levels up to CHF 2500 for adolescents and adults. Beyond the deductible, there is a 10% copayment with a maximum spending limit of CHF 700 annually. The basic insurance coverage encompasses a comprehensive range of inpatient and outpatient services, including pharmaceutical products. Regarding VBID, the Swiss health insurance law permits the differentiation of copayments for drugs with identical active ingredients, allowing a 20% copayment for more expensive drugs [19]. For further details on the Swiss health system, see Schmid et al. [20].

In the current policy debate, VBID implementation in the Swiss health insurance cost-sharing options is considered an alternative to current health plans to enhance access to and use of high-value care, ultimately curbing health cost growth. For instance, the Swiss Parliament has recently suggested zero cost-sharing for effective preventive care such as vaccines [16]. In this context, information provision as a means to direct preferences, e.g., campaigns, is relevant as Switzerland’s strong direct democracy will likely challenge a more systematic implementation of VBID as an alternative to the current system [21]. Such campaigns are real-life examples of how information provision could influence individuals’ preferences and decision-making processes, further emphasizing the importance of studying the impact of information provision on VBID within the Swiss health insurance system.

Our study contributes to the literature in several ways. First, we investigate the impact of information provision on health insurance preferences, specifically focusing on VBID. We combine a DCE with an information experiment in a representative population survey, which, to the best of our knowledge, is a novel approach in the context of the VBID literature. DCEs are state-of-the-art in eliciting individuals’ preferences for products not yet traded in the market, described here by health plan attributes related to VBID. Moreover, we use health insurance premiums as a monetary attribute to derive individuals’ willingness-to-pay (WTP) for VBID elements, which offers a benchmark for cost-savings that would have to be realized in case of a more comprehensive implementation of VBID in the Swiss system. Second, the current discussions on health system reform allow this study to employ a reality-based-DCE design to elicit individuals’ preferences and provide insights into how information provisions, such as text or tabular form, can potentially influence individuals’ decision-making and preferences for specific attributes within current insurance plans. Overall, the findings of this study offer valuable insights to enhance the effectiveness of health insurance decision-making, support ongoing discussions and reforms in the Swiss health insurance system, and ultimately improve our understanding of individuals’ preferences for different VBID elements.

## Methods

### DCE design

DCEs are a popular method for inferring the preferences of individuals. Typically, respondents are presented with a set of attributes related to different (hypothetical) choice options, inferring their preferences from the choices they make based on the information provided. Moreover, DCEs have been combined with information treatments, varying the information provided to individuals before the DCE to assess how preferences are affected. Previous studies employing DCEs have examined the impact of various types of information on consumers’ WTP for different products [22, 23] or the acceptance of specific job offers [24]. We add to this literature in the health domain by using a DCE to measure individuals’ preferences and WTP for VBID attributes.

Table 1 presents the attributes and their corresponding levels to be tested in our DCE. Attributes were selected based on the literature [25] and expert consultations. The yearly deductible, yearly copayment, and disbursement of medicines proved to be highly relevant features when choosing a health plan. Further, these attributes can incorporate value-based incentives. The monthly premium acts as the price attribute.

Levels of the selected attributes were chosen based on the current health system reform debate in Switzerland, and we exchanged them with an expert panel to ensure the realism of each level. Status quo levels reflect the current regulations on health insurance plans in Switzerland. We incorporate value-based incentives in the yearly deductible based on suggested levels by Swiss health insurers (CHF 5000) that we interpret to reduce the use of the least cost-effective care. On the other hand, the Swiss Parliament suggested implementing zero-cost sharing for preventive care [26]. We test these levels through a zero-copayment and zero-deductible. Finally, the Swiss health insurance law allows for a differentiation of copayments for drugs where multiple products with the same active ingredient are available; in this case, copayments can be 20% on the more expensive drugs [27]. More generally, we may interpret the introduction of differentiated copayments and stricter disbursement of medicines based on value as VBID incentives, while the zero- or high-deductible plans can also be understood to reflect current discussions on the general effectiveness of deductibles in reducing moral hazard [28].

**Table 1:** Discrete choice experiment: attributes of health insurance plans and levels

Attributes	Levels	
Yearly deductible	Status quo	Current deductible: CHF 300, 500, 1000, 1500, 2000, or 2500
	Alternatives	CHF 0 or 5000
Yearly copayment	Status quo	10% with a maximum of CHF 700
	Alternatives	0% for services with a high benefit compared to their costs; 10% with a maximum of CHF 700 otherwise. 20% for services with a low benefit compared to their costs up to CHF 1400; 10% with a maximum of CHF 700 otherwise.
Disbursement of medicines	Status quo	According to the current list of disbursed medicines
	Alternative	When several medicines are available, only the one with the highest benefit compared to its cost is reimbursed.
Monthly premium	Status quo	Your current monthly premium
	Alternatives	Increase in monthly premium by CHF 25 or 50 Decrease in monthly premium by CHF 25 or 50

Note: CHF = Swiss franc

The choice tasks in the DCE were generated in the software Ngene [29] using a D-efficient design. Thirty-two choice tasks were broken into four blocks of eight choice tasks to balance DCE complexity and richness of the data to study individuals’ preferences. Choice tasks and alternative orders were randomized to prevent order effects [22], and one choice task was randomly selected as a ninth choice task to check consistency. Each of the four blocks was pilot-tested with a seed sample of 600 individuals to assess whether the choice tasks were clear and that there were no dominant alternatives. No changes in the DCE design were needed, so we included the pilot data in our final dataset. More information about the block design is available in the Appendix 1.

**Table 2:** Example of a choice card

	Model A	Model B
Disbursement of medicines	According to the current list of disbursed medicines	When several medicines are available, only the one with the highest benefit compared to its cost is reimbursed
Yearly copayment	10% with a maximum of CHF 700 per year	10% with a maximum of CHF 700 per year
Yearly deductible	Your current yearly deductible	CHF 5000
Monthly premium	Your current monthly premium	CHF 50 lower than your current premium
**Your choice**:		

Each choice task within the DCE comprised a pair of health insurance plans, as shown in Table 2. Model A represents the respondent’s current health insurance plan (status quo). Model B represents the alternative (hypothetical) plan. Model B differs from Model A by one or two attribute levels, as shown in Table 1, to reduce design complexity. While this would allow us to include two-level interactions in the analysis, they were statistically insignificant; therefore, we do not discuss them further here. Given our specific research interest, we deliberately excluded an opt-out alternative from the DCE, as it could potentially limit the usefulness of the data. Instead, we included the respondent’s current health plan as the reference option [30].

### Experimental treatments

Respondents were randomly assigned to a control group or two treatment groups before taking the DCE to examine the impact of different information provisions. In the control group (*n* = 2024), prior to taking the DCE, respondents could read simple information about the composition of a health insurance plan and ongoing reforms. This information was deemed sufficient to understand the DCE and the attributes used in the choice tasks. In line with the HIL literature [18], treatment group 1 (*n* = 2000) received the same information as the control group, but an additional table with more detailed information about each DCE attribute and their levels, explaining the background and mechanisms related to each attribute was included. This extra information should keep preferences the same as respondents understand basic insurance concepts. Treatment group 2 (*n* = 2009) further received information about the benefits of value-based incentives. Again, this information should not affect preferences if individuals know value-based principles. The information provided to each group is shown in Appendix 2 in the original French, German, and Italian versions, as well as an English support translation.

### Survey and sample

The 2021 Swiss Health Insurance Literacy Survey incorporated the DCE and information experiment. The survey gathered responses from 6033 participants between the ages of 26 and 75, residing in Switzerland’s German-, French-, and Italian-speaking regions. Data collection was outsourced to intervista AG, a private market research company that adheres to the General Data Protection Law and the Federal Act on Data Protection in Switzerland. Members of the online panel provided their general consent to participate in surveys, and they can choose to participate or not in any specific survey. No identification of persons in the data provided by intervista AG is possible. Therefore, according to Art. 2 of the Swiss Human Research Act and the corresponding regulations defined in the Human Research Ordinance, no ethical approval for our study is required, as confirmed by the Ethics Committee Northwestern and Central Switzerland (EKNZ, https://www.eknz.ch).

The questionnaire was translated from English into German, French, and Italian by two native speakers for each language, following the guidelines provided by Epstein et al. [31]. The sample was based on a random draw from intervista’s online panel with over 120,000 actively recruited persons. To ensure the representativeness of the sample for the Swiss population, quotas for gender, age, region, and education were used. Due to the oversampling of certain groups, especially those in the Italian-speaking region, we employed sample weights in all our analyses.

We use several background characteristics from the survey to check for balance across treatment groups, which serves as a test for successful randomization. These include sociodemographic characteristics (age, gender, nationality, monthly income, education), health-related variables (number of doctor visits in the year prior to the interview, chronic health conditions), financial risk aversion (based on the question “Would you consider yourself a person who is fully prepared to take risks, or do you try to avoid risk?” [32]) and time preference (based on the question “Would you consider yourself a person who is fully prepared to give up something today and benefit from it in the future?” [33]) due to the time dimension in the Swiss health insurance market [34, 49], respondents’ current health insurance (deductible level, health plan type), out-of-pocket health expenditures, and HIL; for further details on the data and questionnaire, see [35].

### Empirical specifications

Our statistical analysis is based on a mixed logit model to account for the likely heterogeneity in preferences for VBID elements [36]. The mixed logit incorporates random individual-specific parameters, exploring the within-variation in the data through the repeated choices made by individuals [37]. Following the framework proposed by Hole and Kolstad [38], individual utilities underlying the choices were specified as follows:

1 $$\:{U}_{ijt}=\:{\alpha\:}_{i}{p}_{ijt}+{x}_{ijt}^{{\prime\:}}{\beta\:}_{i}+\:{\epsilon\:}_{ijt};\:\:\:j=A,B;\:\:\:\:t=\text{1,2},\:\dots\:,9\:\:\:\:\:\:$$

where $\:{\alpha\:}_{i}$ and $\:{\beta\:}_{i}$ denote the individual-specific preference parameters associated with the different choice attributes. The variable $\:{p}_{ijt}$ represents the level of the premium attribute for individual *i* in model (choice option) *j* at choice task *t*, while the vector $\:{x}_{ijt}$ represents the levels of the other health plan attributes, and $\:{\epsilon\:}_{ijt}$ is a random error following a type-I extreme value distribution. The random utility framework assumes that individuals choose that option *j* (*A* or *B*) in choice task *t* that gives them the higher utility $\:{U}_{ijt}$, completing the model.

Unlike contingent valuation studies [39], we derived the WTP indirectly from the preference space in Eq. [1]. This regime yielded a better fit than estimation in the WTP space, which has been discussed as an alternative in the literature [38]. In the estimation, we specified$\:{\:-\alpha\:}_{i}$ to follow a log-normal distribution as individuals were assumed to derive a negative utility from increasing premiums. The other parameters of the model were assumed to be normally distributed, and all random parameters were allowed to be correlated. The vector of attributes included an alternative-specific constant to control for general preferences for the status quo instead of the alternative (hypothetical) health plan; see de Bresser et al. [40] for a related approach to derive the WTP for in-kind and in-cash home care insurance. Note that there is no need to include individual-specific background characteristics in the estimation of (1) due to the design, i.e., attribute levels are assigned independently of any characteristics of the individuals, and estimation explores the within-individual variation. Based on the individual-level predictions of the parameters in the model, we ran regression-based tests to see if estimates were different across treatment arms.

## Results

### Sample description and balance tests

Socio-demographics, health characteristics, and health insurance choices for the total sample and per treatment arm are presented in Table 3. The distribution of respondents across language regions is as expected [29]. All respondents are Swiss residents, and most have Swiss nationality (91.2%). Thirty-six percent completed tertiary education, as expected [30]. On average, respondents were 49.6 years old (SD = 13.74). They reported an average of 3.6 doctor visits the year before the interview (SD = 6.98), and most were not suffering from a chronic illness. Regarding health insurance, most of the sample chose a yearly deductible of CHF 300 or 2500, again as expected; 55.1% had a family doctor health plan, and the median out-of-pocket spending was less than CHF 500.

Table 3 also reports a composite measure of HIL [35]. The mean value of 2.86 (SD = 0.52) shows that, on average, HIL is moderate with significant dispersion [41]. Further, respondents tend to be financially risk-averse and more forward-looking. Most importantly, statistics in the last column of Table 3 confirm balanced background characteristics across treatment arms, which is reassuring regarding our experimental design.

**Table 3:** Background characteristics overall and by treatment group and balance tests

	Pooled	Control group	Treatment group1	Treatment group 2	Balance tests
Female	50.04	49.32	50.30	50.51	0.740
Age†	49.61 (13.74)	49.69 (13.87)	49.57 (13.75)	49.58 (13.59)	0.232
Non-Swiss	8.83	8.79	7.94	9.76	0.129
Tertiary education	36.29	35.95	37.61	35.32	0.295
Monthly income in CHF^a^					0.932
< 4500	18.52	18.53	18.52	18.51	
4500–5999	19.27	19.88	19.40	18.52	
6000–8999	30.67	30.82	31.08	30.10	
≥ 9000	31.55	30.73	31.00	32.87	
Number of doctor visits†	3.62 (6.98)	3.66 (9.81)	3.70 (7.05)	3.52 (7.08)	0.455
Chronic health condition^b^	37.88	38.65	38.70	39.67	0.373
Type of health plan^c^					0.863
Basic	19.30	19.48	19.68	18.73	
HMO	8.95	8.89	8.42	9.54	
Telemedicine	16.65	16.82	17.07	16.07	
Family doctor	55.10	54.80	54.84	55.65	
Yearly deductible in CHF^d^					0.832
300	38.35	39.13	38.73	40.20	
500	10.16	10.26	10.65	9.57	
1 000	3.73	3.50	3.90	3.81	
1 500	6.60	7.05	5.86	6.89	
2 000	3.66	3.42	3.99	3.58	
2 500	36.49	36.65	36.87	35.96	
Out-of-pocket health expenditures in CHF^e^					0.740
None	10.10	10.39	10.75	10.86	
1–299	25.57	27.71	27.64	25.70	
300–499	19.62	20.45	19.68	22.07	
500–999	20.12	20.83	20.98	21.96	
1000–1499	9.96	10.84	10.79	9.95	
≥ 1500	9.27	9.78	10.17	9.44	
Linguistic region					0.336
German	70.82	70.24	70.52	71.69	
French	23.33	24.22	23.87	21.89	
Italian	5.86	5.54	5.61	6.42	
Health insurance literacy†	2.86 (0.52)	2.86 (0.51)	2.85 (0.53)	2.86 (0.53)	0.104
Financial risk-taking†	2.39 (1.2)	2.38 (18)	2.42 (1.22)	2.39 (1.21)	0.603
Time preferences†	3.40 (1.16)	3.41 (1.17)	3.42 (1.17)	3.38 (1.15)	0.852
Number of individuals	6033	2024	2000	2009	

*Source*: 2021 Swiss Health Insurance Literacy Survey. *Notes*: Reported numbers are weighted sample proportions, or sample means and standard deviations (in brackets) for variables marked with †, in total and per treatment arm (control, treatment groups 1 and 2). Weights were used to reflect oversampling for selected population groups. Income was missing for 17.47% of the sample (1053 individuals). Number of doctor visits in the last 12 months; results do not display 26 individuals who did not answer. Type of health plans: “basic” refers to free choice of providers; managed care plans composed of HMO, telemedicine, or family doctor; category “other” with 1.77% of the sample who did not want to report it. Number of responses in category “don’t know”, “no answer”, or “other” not shown for the variables (a) 17.47%/17.38%/17.74%/17.29%, (b) 2.07%/2.05%/1.94%/2.08%, (c) 4.36%/4.08%/4.95%/4.60%, (d) 1.94%/1.90%/2.14%/1.78%, and (e) 5.36%/5.43%/5.56%/5.09% (per column). Health insurance literacy on a scale from 1 = very bad to 4 = very good. Willingness to take financial risks and willingness to sacrifice something today to benefit in the future (time preferences) on a scale from 1 = completely unwilling to 5 = completely willing. The last column shows *p*-values for chi-squared tests of the null hypothesis of equal distributions of the characteristics between the treatment and control groups

### Mixed logit estimates and WTP for VBID

Table 4 shows the estimated coefficients of the mixed logit models and the estimated WTP for VBID elements per treatment arm. Overall, respondents show a clear preference for the status quo, i.e., with the choice options presented in the DCE, respondents would be willing to choose an alternative health plan over their current plan if monthly premiums would decrease by CHF 46.44, on average, or more compared to the status quo. The preference for the current plan decreases, or vice versa - there is less opposition against the VBID alternatives - once additional information about the insurance attributes, particularly VBID, is shown to the respondents (*p* < 0.01).

Regarding the specific VBID attributes, providing additional information about the mechanisms of the insurance attributes reduces the opposition against stricter disbursement of medicines based on value. However, such a restriction per se still reduces respondents’ utilities, on average, and therefore comes with a negative mean WTP. The information treatments also seem to anchor preferences, e.g., with the explanation of the 10% copayments (see Appendix 2). Consistent with such anchoring effects, we find that respondents have lower preferences for a 0% copayment on high-value care but, at the same time, dislike a 20% copayment, even if this refers to low-value care only. Similarly, preferences for a zero-deductible plan are also reduced with the information treatments, while there is a stronger preference against a new high-deductible plan at CHF 5000 per year.

The marginal utility of higher monthly premiums is negative and does not differ between treatment arms. The latter result is noteworthy because our information treatments included a more detailed explanation of VBID attributes as well as an explanation of the monthly premium, but the latter part did not seem to have influenced respondents’ preferences, likely because respondents are familiar with this aspect of health insurance. On the other hand, more complex elements of health insurance plans, such as copayments, deductibles, or reimbursements of medicines, may be more difficult to understand, and thus, providing more detailed explanations of these concepts affects the preferences of individuals for health plans that give more emphasis on VBID attributes.

**Table 4:** Mixed logit estimates and WTP per treatment arm

		Control group		Treatment group 1		Treatment group 2				
		*β*	*WTP*	*β*	*WTP*	*β*	*WTP*	0 = 1	0 = 2	1 = 2
***Mean estimates***										
Model B (alternative to status quo)		-1.591	*-46.44*	-1.427	*-40.27*	-1.259	*-35.49*	< 0.01	< 0.01	< 0.01
		(0.145)	*(4.61)*	(0.143)	*(4.37)*	(0.143)	*(4.41)*			
*VBID elements*										
Copayment	0% high-value care	0.567	*16.55*	0.320	*9.04*	0.321	*9.04*	< 0.01	< 0.01	0.92
		(0.110)	*(3.19)*	(0.103)	*(2.89)*	(0.103)	*(2.88)*			
	20% low-value care	-0.989	*-28.86*	-1.283	*-36.22*	-1.295	*-36.48*	< 0.01	< 0.01	0.77
		(0.149)	*(4.26)*	(0.154)	*(4.26)*	(0.150)	*(4.141)*			
Drug disbursement based on value		-0.909	*-26.54*	-0.709	*-20.01*	-0.748	*-21.08*	< 0.01	< 0.01	< 0.01
		(0.138)	*(3.95)*	(0.140)	*(3.99)*	(0.132)	*(3.71)*			
*General cost-sharing*										
Deductible	CHF 0	1.176	*34.34*	0.936	*26.43*	1.169	*32.94*	< 0.01	0.39	< 0.01
		(0.098)	*(3.01)*	(0.098)	*(2.92)*	(0.093)	*(2.87)*			
	CHF 5000	-4.144	*-120.99*	-4.165	*-117.6*	-4.871	*-137.3*	0.88	< 0.01	< 0.01
		(0.264)	*(7.18)*	(0.268)	*(7.05)*	(0.320)	*(8.13)*			
Monthly premium		-0.034		-0.035		-0.035		0.31	0.11	0.63
		(0.002)		(0.002)		(0.002)				
***Standard deviation estimates***										
Model B (alternative to status quo)		1.781		1.780		1.950		0.99	0.90	0.90
		(0.081)		(0.085)		(0.084)				
*VBID elements*										
Copayment	0% high-value care	0.501		0.912		0.773		0.46	0.63	0.68
		(0.507)		(0.217)		(0.259)				
	20% low-value care	1.492		1.460		1.680		0.88	0.33	0.30
		(0.146)		(0.167)		(0.130)				
Drug disbursement based on value		0.810		0.762		0.617		0.89	0.51	0.63
		(0.230)		(0.248)		(0.178)				
*General cost-sharing*										
Deductible	CHF 0	1.324		1.562		1.454		0.31	0.55	0.60
		(0.173)		(0.158)		(0.132)				
	CHF 5000	2.877		2.972		3.078		0.76	0.54	0.74
		(0.222)		(0.210)		(0.245)				
Monthly premium		0.029		0.039		0.037		0.05	0.17	0.75
		(0.003)		(0.004)		(0.005)				
Log-likelihood value		-7084.5		-7084.52		-7247.55				
Number of individuals		2024		2000		2009				

*Source*: Swiss Health Insurance Literacy Survey 2021. *Notes*: The table shows the results of mixed logit models for the choice of model B vs. A using the nine choices per individual included in the experiment in each treatment arm. Explanatory variables include the attributes listed in Table 1 and a preference parameter for model B. The mixed logit model allows for individual-level variation in the coefficients, assuming a normal distribution for each attribute’s coefficient (estimated by *mixlogit* in Stata). In block 1, the *β* column shows the estimated mean coefficients of the random parameters. The WTP column reports the negative ratio of the mean coefficient of the attribute and the mean coefficient of the monthly premium to derive the willingness-to-pay. In block 2, the *β* column shows the standard deviation estimates of the random parameters. Standard errors are shown in parentheses for all estimates. *P*-values of Wald tests for the null hypothesis of no differences between the treatment arms are reported in the last three columns of the table (control group = 0, treatment group 1 = 1, treatment group 2 = 2) based on the posterior estimates of the individual-level coefficients and standard deviations after the mixed logit estimations per arm

Regarding the heterogeneity in the distribution of preferences, the standard deviation estimates reported in Table 4 indicate significant variability in the random individual-specific parameters in general and regarding the specific VBID attributes. However, the results show that the preference heterogeneity does not differ between the control and treatment groups. Thus, the information treatments only led to a level shift in preferences but did not change the variability in preference distributions, which could have been interpreted as a change in individuals’ uncertainty about the different VBID attributes. Instead, individuals seem to be less opposed to (or more inclined to choose) alternative health plans with VBID features once they are more aware of their mechanisms.

To complement the results reported in Table 4, we obtained the posterior estimates of the individual-level coefficients from the mixed logit regressions by treatment group [38]; see also Appendix 3 for the distribution of the individual-level WTP estimates derived from the coefficients. As these posteriors are estimated, their mean values reported in Table 5 (in italics, column *All*) are close but not identical to the estimates reported in Table 4. We then used linear regressions to estimate the average treatment effects (ATEs) for the two information treatments (reported in relative terms) for the overall sample and separately by individuals with a low and a high HIL to explore our conjectured mechanism of limited HIL in the population. The results in Table 5 suggest that individuals with low HIL indeed respond more to the provision of insurance information compared to individuals with high HIL. This is true in terms of general preferences for the alternative health plans shown in our DCE, but also for specific VBID elements by reducing the opposition against stricter disbursement of medications based on value as well as the anchoring effects of the 10% copayments.

To this end, it is essential to note the different levels of preferences, with low HIL individuals showing more vigorous opposition, on average, against alternative health plans. Such level shifts are also found concerning individuals’ healthcare needs. For example, respondents with chronic health conditions are more opposed to alternative health plans, have stronger preferences for zero copayments for high-value care or zero deductibles, and have lower preferences for higher cost-sharing or attributes with access restrictions, as depicted in Table 6. On the other hand, we do not find significant differences in the effects of the information treatments along this dimension of background characteristics, which is an interesting result for two reasons. First, one might suspect that the experience of using health insurance increases with chronic health conditions due to exposure to the health system and, relatedly, familiarity with health insurance concepts such as copayments and deductibles. However, in complementary analyses, we found that after controlling for basic demographic characteristics, such as gender and age, the association between chronic health conditions and the HIL score is small and statistically insignificant. Second, value-based principles might benefit the chronically ill more as they have more intensive encounters with the health system. Thus, by making them more aware of such principles (treatment 2), one could have expected higher preferences in favor of related attributes, which does not seem to be the case. The latter result is not specific to chronic health conditions, and the same pattern is observed for other characteristics describing high needs, e.g., the number of doctor consultations or out-of-pocket health expenditures. Potential explanations for this pattern are that HIL is indeed the decisive factor in forming preferences, or the value of the information treatments per se is low, or it is understood differently by different groups of the population, e.g., by HIL levels. While it was not our goal to compare different forms of information provision in this study, future research should investigate such patterns in greater detail by aiming to improve individuals’ understanding of VBID principles and their mechanisms and explain the involved trade-offs for individuals when choosing a specific health plan.

**Table 5:** Average posteriors of individual-level coefficients and relative treatment effects by HIL subgroup

			All			Low HIL			High HIL	
			Treatment 1	Treatment 2		Treatment 1	Treatment 2		Treatment 1	Treatment 2
Model B (alternative to status quo)			*-1.587*			*-1.614*			*-1.411*	
			+ 9.3%	+ 19.6%		+ 19.4%	+ 25.7%		+ 7.9%	+ 12.9%
			(2.4%)	(2.5%)		(7.3%)	(7.6%)		(7.2%)	(7.5%)
*VBID elements*										
Copayment	0% high-value care		*0.570*			*0.561*			*0.572*	
			-44.2%	-44.1%		-45.0%	-44.8%		-38.2	-40.2%
			(1.2%)	(0.8%)		(3.5%)	(2.5%)		(3.8%)	(3.2%)
	20% low-value care		*-0.982*			*-0.941*			*-1.002*	
			-30.6	-31.5%		-36.9	-46.3%		-29.9%	-28.8%
			(2.7%)	(3.0%)		(9.5%)	(10.1%)		(8.1%)	(8.9%)
Drug disbursement based on value			*-0.909*			*-0.919*			*-0.925*	
			+ 21.8%	+ 17.7%		+ 25.1%	+ 20.5%		+ 21.4%	+ 17.3%
			(0.8%)	(0.7%)		(2.4%)	(2.2%)		(2.3%)	(2.0%)
*General cost-sharing*										
Deductible	CHF 0		*1.178*			*1.141*			*1.152*	
			-20.6%	-1.3%		-14.4%	+ 6.3		-25.2%	-8.3%
			(1.8%)	(1.8%)		(5.7%)	(6.1%)		(5.3%)	(5.4%)
	CHF 5000		*-4.178*			*-4.198*			*-4.238*	
			+ 0.0	-16.6%		+ 6.4	-12.9%		-1.6	-16.9%
			(1.1%)	(1.3%)		(4.0%)	(4.3%)		(3.3%)	(3.7%)
Monthly premium			*-0.034*			*-0.037*			*-0.033*	
			-2.8	-3.4%		-3.6%	-2.7%		-2.5	-2.6%
			(1.9%)	(1.9%)		(5.9%)	(6.3%)		(6.3%)	(6.4%)

*Source*: Swiss Health Insurance Literacy Survey 2021. *Notes*: The table shows the mean values of the posterior estimates of the individual-level coefficients after the mixed logit regressions for the control group in italics (baseline values) for the overall sample (*n* = 6033), a subsample with low HIL (bottom 10% of the HIL scale; see (35) for details on the construction of the scale; *n* = 594), and a subsample with high HIL (top 10% of the HIL scale; *n* = 610). Treatment effects are estimated using linear regressions with robust standard errors from the posterior estimates using the data from the control group and the two treatment groups (as shown in Table 4), overall and by HIL subsample. Since baseline preferences vary by subsample, treatment effects are reported in percent values relative to the baseline estimates for better comparability. Standard errors (in parentheses) are calculated using the delta method from the estimated baseline and treatment effects in the linear regressions

**Table 6:** Average posteriors of individual-level coefficients and relative treatment effects by health status

			All			No chronic health condition			Chronic health condition	
			Treatment 1	Treatment 2		Treatment 1	Treatment 2		Treatment 1	Treatment 2
Model B (alternative to status quo)			*-1.587*			*-1.405*			*-1.865*	
			+ 9.3%	+ 19.6%		+ 8.6%	+ 22.0%		+ 9.7%	+ 17.8%
			(2.4%)	(2.5%)		(3.6%)	(3.6%)		(3.1%)	(3.3%)
*VBID elements*										
Copayment	0% high-value care		*0.570*			*0.473*			*0.719*	
			-44.2%	-44.1%		-44.0	-44.3%		-44.3%	-44.1%
			(1.2%)	(0.8%)		(1.5%)	(1.2%)		(1.8%)	(1.3%)
	20% low-value care		*-0.982*			*-0.929*			*-1.068*	
			-30.6	-31.5%		-31.9%	-29.5%		-28.3	-33.2%
			(2.7%)	(3.0%)		(3.9%)	(4.2%)		(3.7%)	(4.1%)
Drug disbursement based on value			*-0.909*			*-0.891*			*-0.933*	
			+ 21.8%	+ 17.7%		+ 21.8%	+ 16.7%		+ 21.7%	+ 19.0%
			(0.8%)	(0.7%)		(1.0%)	(0.9%)		(1.2%)	(1.0%)
*General cost-sharing*										
Deductible	CHF 0		*1.178*			*1.096*			*1.233*	
			-20.6%	-1.3%		-20.9%	-2.2%		-20.1%	+ 0.1%
			(1.8%)	(1.8%)		(2.3%)	(2.3%)		(3.0%)	(3.0%)
	CHF 5000		*-4.178*			*-4.090*			*-4.317*	
			+ 0.0	-16.6%		+ 0.1	-16.3%		-0.1	-17.3%
			(1.1%)	(1.3%)		(1.7%)	(1.8%)		(1.6%)	(1.7%)
Monthly premium			*-0.034*			*-0.036*			*-0.032*	
			-2.8	-3.4%		-4.4%	-3.4%		-1.3	-3.3%
			(1.9%)	(1.9%)		(2.7%)	(2.6%)		(2.6%)	(2.7%)

*Source*: Swiss Health Insurance Literacy Survey 2021. *Notes*: The table shows the mean values of the posterior estimates of the individual-level coefficients after the mixed logit regressions for the control group in italics for the overall sample (*n* = 6033), a subsample without chronic health condition (*n* = 3623), and a subsample with chronic health condition (*n* = 2285); see also Table 3. Treatment effects are estimated using linear regressions with robust standard errors from the posterior estimates after the mixed logit regressions for the control group and the two treatment groups (as shown in Table 4), overall and by subsample with and without chronic health condition. Since baseline preferences vary by subsample, treatment effects are reported in percent values relative to the baseline estimates for better comparability. Standard errors (in parentheses) are calculated using the delta method from the estimated baseline and treatment effects in the linear regressions

## Discussion

This study delves into individuals’ preferences for VBID attributes, assessing whether providing additional information can change their preferences for health plans with VBID elements. The control group’s results revealed a strong preference of participants for their current plan as opposed to the alternatives presented in the DCE. However, providing more detailed information on the mechanisms of health insurance and value-based principles, as in our information experiment, showed a significant increase in preferences for alternative health plans with VBID elements, indicating the importance of HIL in health plan choices.

It is essential to acknowledge certain limitations in this study. First, as mentioned above, we focused on the impact of information provision rather than the information *per se*, quality, and presentation format. Future research should explore the effectiveness of different types of information as the literature suggests, for example, that graphical information may be better understood than text-based information [13, 18, 42]. Additionally, although our experiment environment is unique, its restricted focus might limit replicability, requiring proper contextual adaptations. Second, using a DCE to measure preferences comes with the usual constraints, such as hypothetical bias, limited scope on the range of included attributes and levels, and the trade-offs between design complexity and cognitive burden on respondents. While we pilot-tested the survey and allowed for multiple feedback loops with experts and practitioners in the health insurance field, we must consider that results would look different when adding additional attributes and levels. Against that backdrop, further research is granted to understand preferences for VBID, e.g., using alternative designs for the DCE or other study designs [43]. Third, the sample is based on intervista’s online panel. While the panel is actively maintained and quality assurance mechanisms are in place to make the panel representative of the Swiss population, including hard-to-reach populations, it is restricted, by its nature, to the internet-using population. We restricted our sample to individuals aged 26–75 for the same reasons. Therefore, certain population groups could not be included in our study, and further research is needed to expand the scope of preference assessments to younger and older populations or those on the margin, which are likely not represented in intervista’s online panel. Finally, using mixed logit regressions comes with the usual constraints, and further studies may investigate different assumptions on preference heterogeneity, which may be refined in terms of shape and scale [44].

Despite these limitations, we believe that the implications of our research extend beyond the study’s scope and hold relevance for Switzerland’s health insurance landscape more generally, with potential applications also in other choice-based health insurance systems, such as the Netherlands, Germany, or the US Health Insurance Marketplace^®^. Our findings emphasize the importance of strategically communicating information about VBID, with the potential to receive widespread support for VBID reform plans if VBID holds its promise of generating more value for the money that ultimately can help secure cost savings for consumers.

Future research could explore further the relationship between HIL and health insurance information provision. Moreover, examining sub-samples based on geographical regions would be beneficial, as Switzerland’s cultural differences across language regions, as well as urban-rural differences, may impact health insurance preferences significantly [45–47]. This likely requires additional data collection since a mixed logit model requires a relatively large sample, which is challenging to achieve for smaller geographic areas. However, such an exploration could provide valuable insights into tailoring communication strategies in light of limited HIL [41, 48] and VBID implementation approaches in different linguistic and cultural contexts.

In conclusion, this study sheds light on individuals’ preferences for VBID and the significance of health insurance information provision. The results highlight the need for targeted communication to promote the adoption of VBID and increase individuals’ understanding of health insurance. Despite its limitations, our study contributes to the literature on health insurance decision-making as it is the first to study the impact of information on preferences for VBID. Our work has broader implications for reforms in choice-based health insurance systems. By understanding the impact of information provision on preferences, policymakers can design more effective interventions to improve health plan choices in Switzerland and other countries with similar insurance systems.

## Electronic supplementary material

Below is the link to the electronic supplementary material.

Supplementary Material 1

Supplementary Material 2

Supplementary Material 3

## Data Availability

The data that support the findings of this study are available free of charge upon request after signing a data contract with the Center for Health, Policy and Economics (CHPE) at the University of Lucerne, Switzerland. Contact by email via chpe@unilu.ch with a brief description of the planned research and dissemination of results. Restrictions apply to the availability of data that are part of a broader study and provided by intervista AG. Data users may gain access to datasets only after accepting an agreement to use and cite the data in a proper fashion, for scientific research and education within an academic framework, and following typical scientific, ethical norms of conduct. However, all datasets will be available from the corresponding author upon reasonable request.
