# Supplementary material for: Does targeted information impact consumers’ preferences for value-based health insurance? Evidence from a survey experiment
Source: Health Econ Rev. 2024 Nov 18;14:94. doi: 10.1186/s13561-024-00573-9 (PMC11571679; doi:10.1186/s13561-024-00573-9)
Supplement: Supplementary file 1 — Supplementary Material 1 [file 13561_2024_573_MOESM1_ESM.docx]

Table A1. DCE blocks overview

| **Status quo (Model A)** | | | | **Alternative (Model B)** | | | | **Block** |
| --- | --- | --- | --- | --- | --- | --- | --- | --- |
| **Drugs** | **Copay** | **Deductible** | **Premium** | **Drugs** | **Copay** | **Deductible** | **Premium** |  |
| 0 | 0 | 0 | 0 | 1 | 0 | 1 | 50 | 1 |
| 0 | 0 | 0 | 0 | 1 | 0 | 1 | -50 | 1 |
| 0 | 0 | 0 | 0 | 0 | 1 | 5000 | 0 | 1 |
| 0 | 0 | 0 | 0 | 1 | 0 | 1 | 0 | 1 |
| 0 | 0 | 0 | 0 | 1 | 0 | 5000 | -50 | 1 |
| 0 | 0 | 0 | 0 | 1 | 1 | 1 | 25 | 1 |
| 0 | 0 | 0 | 0 | 1 | 1 | 0 | 50 | 1 |
| 0 | 0 | 0 | 0 | 0 | 2 | 0 | -25 | 1 |
| 0 | 0 | 0 | 0 | 0 | 2 | 5000 | -25 | 2 |
| 0 | 0 | 0 | 0 | 0 | 2 | 1 | 50 | 2 |
| 0 | 0 | 0 | 0 | 0 | 2 | 1 | 25 | 2 |
| 0 | 0 | 0 | 0 | 1 | 0 | 0 | -50 | 2 |
| 0 | 0 | 0 | 0 | 0 | 1 | 1 | 50 | 2 |
| 0 | 0 | 0 | 0 | 0 | 1 | 5000 | 50 | 2 |
| 0 | 0 | 0 | 0 | 0 | 2 | 5000 | -50 | 2 |
| 0 | 0 | 0 | 0 | 0 | 2 | 1 | 0 | 2 |
| 0 | 0 | 0 | 0 | 1 | 0 | 0 | -25 | 3 |
| 0 | 0 | 0 | 0 | 0 | 2 | 5000 | 0 | 3 |
| 0 | 0 | 0 | 0 | 1 | 2 | 0 | -50 | 3 |
| 0 | 0 | 0 | 0 | 0 | 1 | 5000 | -25 | 3 |
| 0 | 0 | 0 | 0 | 1 | 0 | 5000 | -25 | 3 |
| 0 | 0 | 0 | 0 | 0 | 1 | 5000 | -50 | 3 |
| 0 | 0 | 0 | 0 | 1 | 1 | 0 | 25 | 3 |
| 0 | 0 | 0 | 0 | 0 | 2 | 1 | -25 | 3 |
| 0 | 0 | 0 | 0 | 0 | 2 | 0 | 0 | 4 |
| 0 | 0 | 0 | 0 | 1 | 0 | 1 | 25 | 4 |
| 0 | 0 | 0 | 0 | 1 | 1 | 0 | 0 | 4 |
| 0 | 0 | 0 | 0 | 1 | 0 | 5000 | 0 | 4 |
| 0 | 0 | 0 | 0 | 0 | 1 | 5000 | 25 | 4 |
| 0 | 0 | 0 | 0 | 1 | 0 | 0 | 50 | 4 |
| 0 | 0 | 0 | 0 | 0 | 2 | 1 | -50 | 4 |
| 0 | 0 | 0 | 0 | 1 | 0 | 0 | 25 | 4 |

| *Notes.* This table shows the block overview that were selected for the discrete choice experiment.  The numbers correspond to attribute levels as follows:   \| Drugs (disbursement) \| 0 \| According to the current list of disbursed medicines \| \| --- \| --- \| --- \| \| 1 \| When several medicines are available, only the one with the highest benefit compared to its cost is reimbursed \| \| Copay (yearly) \| 0 \| 10% with a maximum of CHF 700 \| \| 1  2 \| 1. 0% for health services with a high value compared to their cost; 10% with a maximum of CHF 700 otherwise 2. 20% for health services with a low value compared to their cost up to CHF 1400; 10% with a maximum CHF 700 otherwise \| \| Deductible (yearly) \| 0 \| Current deductible: CHF 300, 500, 1000, 1500, 2000, or 2500 \| \| 1  5000 \| 1. CHF 0 2. CHF 5000 \| \| Premium (monthly) \| 0 \| Your current monthly premium \| \| -50  -25  25  50 \| 1. Decrease in monthly premium by CHF 50 2. Decrease in monthly premium by CHF 25 3. Increase in monthly premium by CHF 25 4. Increase in monthly premium by CHF 50 \| | | | |
| --- | --- | --- | --- | --- | --- | --- | --- | --- | --- | --- | --- | --- | --- | --- | --- | --- | --- | --- | --- | --- | --- | --- | --- |
|  |  |  |  |
