# Supplementary material for: Does targeted information impact consumers’ preferences for value-based health insurance? Evidence from a survey experiment
Source: Health Econ Rev. 2024 Nov 18;14:94. doi: 10.1186/s13561-024-00573-9 (PMC11571679; doi:10.1186/s13561-024-00573-9)
Supplement: Supplementary file 2 — Supplementary Material 2 [file 13561_2024_573_MOESM2_ESM.docx]

# Context provided to the participants of the DCE

The DCE consists of a series of nine choices (eight different and one randomly repeated choice) between the respondents’ current health insurance (model A) and a hypothetical health insurance incorporating VBID elements (model B). Before taking the DCE, participants were randomly allocated to one of the three information groups introducing the choice tasks. The text below was translated into German, French, and Italian and presented to the respondents, depending on their choice of language before starting the whole survey. The English version was not presented to the participants.

## English version

### 1.1 Group 1

*Please read the following instructions carefully. They are important for the next questions.*

*Various changes to basic insurance are currently being discussed in the context of Swiss health policy. In the following, we will present you with hypothetical models of basic insurance, and we would like you to tell us which ones you prefer.*

*Basic health insurance has the following attributes: the monthly insurance premium, the yearly deductible, the yearly copayment, and the reimbursement of drugs. Based on these attributes, we will present scenarios showing two health insurance models (A and B). Please choose the insurance model that suits you best. Please indicate your spontaneous preference; there are no right or wrong answers.*

### 1.2 Group 2

*Please read the following instructions carefully. They are important for the next questions.*

*Various changes to basic insurance are currently being discussed in the context of Swiss health policy. In the following, we will present you with hypothetical models of basic insurance, and we would like you to tell us which ones you prefer.*

*Basic health insurance has the following attributes*:

| *Reimbursement of drugs* | *Health insurance law specifies that basic health insurance reimburses only effective, appropriate, and inexpensive drugs. These reimbursed drugs are grouped in a list.* |
| --- | --- |
| *Yearly copayment* | *All insured persons must pay 10% of their healthcare costs out-of-pocket, up to a maximum of CHF 700 per year. The co-payment applies to all services (including medication) covered by basic health insurance that exceed the annual deductible.* |
| *Yearly deductible* | *The deductible represents a fixed amount up to which you must pay your own healthcare costs per year. This fixed amount applies to all services (including medicines) covered by basic health insurance.* |
| *Monthly premium* | *The monthly premium is the amount you pay each month to the health insurance company for your basic insurance. The monthly premium for adults (aged 26 and over) depends on the canton of residence, the insurer, the insurance model, and the deductible. In 2020, insurers charged an average monthly premium of around CHF 320 for basic insurance.* |

*Based on these attributes, we will present scenarios showing two health insurance models (A and B). Please choose the insurance model that suits you best. Please indicate your spontaneous preference; there are no right or wrong answers.*

### 1.3 Group 3

*Please read the following instructions carefully. They are important for the next questions.*

*Various changes to basic insurance are currently being discussed in the context of Swiss health policy. In the following, we will present you with hypothetical models of basic insurance, and we would like you to tell us which ones you prefer.*

*Basic health insurance has the following attributes*:

| *Reimbursement of drugs* | *Health insurance law specifies that basic health insurance reimburses only effective, appropriate, and inexpensive drugs. These reimbursed drugs are grouped in a list.* |
| --- | --- |
| *Yearly copayment* | *All insured persons must pay 10% of their healthcare costs out-of-pocket, up to a maximum of CHF 700 per year. The co-payment applies to all services (including medication) covered by basic health insurance that exceed the annual deductible.* |
| *Yearly deductible* | *The deductible represents a fixed amount up to which you must pay your own healthcare costs per year. This fixed amount applies to all services (including medicines) covered by basic health insurance.* |
| *Monthly premium* | *The monthly premium is the amount you pay each month to the health insurance company for your basic insurance. The monthly premium for adults (aged 26 and over) depends on the canton of residence, the insurer, the insurance model, and the deductible. In 2020, insurers charged an average monthly premium of around CHF 320 for basic insurance.* |

*Currently, health insurance reimburses drugs and services that are of little use to patients or are costly with little benefits. A lower copayment for medicines and services that have been proven to provide the greatest patient benefit would help to avoid unnecessary expenditures in the health system and increase the quality of care.*

*Based on these attributes, we will present scenarios showing two health insurance models (A and B). Please choose the insurance model that suits you best. Please indicate your spontaneous preference; there are no right or wrong answers.*

## Version française

### 2.1 Groupe 1

*Veuillez lire attentivement les instructions suivantes, elles sont importantes pour les prochaines questions.*

*Diverses modifications de l'assurance de base sont actuellement discutées dans le cadre de la politique de santé suisse. Dans ce qui suit, nous allons vous présenter des modèles hypothétiques de l'assurance de base et nous aimerions que vous nous disiez ceux que vous préférez.*

*L’assurance maladie de base possède les attributs suivants : la prime d'assurance mensuelle, la franchise annuelle et la quote-part annuelle, ainsi que le remboursement des médicaments. Sur la base de ces attributs, nous allons vous présenter des scenarios montrant deux modèles d'assurance maladie (A et B). Veuillez choisir le modèle d'assurance qui vous convient le mieux. Veuillez simplement indiquer votre préférence spontanée ; il n'y a pas de bonnes ou de mauvaises réponses.*

### 2.2 Groupe 2

*Veuillez lire attentivement les instructions suivantes, elles sont importantes pour les prochaines questions.*

*Diverses modifications de l'assurance de base obligatoire sont actuellement discutées dans le cadre de la politique de santé suisse. Dans ce qui suit, nous allons vous présenter des modèles hypothétiques de l'assurance de base et nous aimerions que vous nous disiez ceux que vous préférez.*

*Le modèle d’assurance maladie de base possède les attributs suivants :*

| *Remboursement des médicaments* | *La loi sur l'assurance maladie stipule que seuls les médicaments efficaces, appropriés et peu onéreux sont remboursés par l'assurance maladie de base. Ces médicaments remboursés sont regroupés dans une liste.* |
| --- | --- |
| *Quote-part annuelle* | *Tous les assurés sont tenus de payer de leur poche 10% de leurs frais de santé, jusqu'à hauteur de 700 CHF par an. La quote-part s'applique à toutes les prestations (y compris les médicaments) couvertes par l'assurance de base qui dépassent la franchise annuelle.* |
| *Franchise annuelle* | *La franchise représente un montant fixe jusqu'à concurrence duquel vous devez payer vous-même vos frais de santé par an. Ce montant fixe s'applique à tous les services (y compris les médicaments) couverts par l'assurance maladie de base.* |
| *Prime mensuelle* | *La prime mensuelle est le montant que vous payez chaque mois à la compagnie d'assurance maladie pour votre assurance de base. La prime mensuelle pour les adultes (âgés de 26 ans et plus) dépend du canton de résidence, de l'assureur, du modèle d'assurance et de la franchise. En 2020, les assureurs ont facturé une prime mensuelle moyenne d'environ 320 CHF pour l'assurance de base.* |

*Sur la base de ces attributs, nous allons maintenant vous présenter des scenarios montrant deux modèles d'assurance maladie (A et B). Veuillez choisir le modèle d'assurance qui vous convient le mieux. Veuillez simplement indiquer votre préférence spontanée ; il n'y a pas de bonnes ou de mauvaises réponses.*

### 2.3 Groupe 3

*Veuillez lire attentivement les instructions suivantes, elles sont importantes pour les prochaines questions.*

*Diverses modifications de l'assurance de base obligatoire sont actuellement discutées dans le cadre de la politique de santé suisse. Dans ce qui suit, nous allons vous présenter des modèles hypothétiques de l'assurance de base et nous aimerions que vous nous disiez ceux que vous préférez.*

*Le modèle d’assurance maladie de base possède les attributs suivants :*

| *Remboursement des médicaments* | *La loi sur l'assurance maladie stipule que seuls les médicaments efficaces, appropriés et peu onéreux sont remboursés par l'assurance maladie de base. Ces médicaments remboursés sont regroupés dans une liste.* |
| --- | --- |
| *Quote-part annuelle* | *Tous les assurés sont tenus de payer de leur poche 10 % de leurs frais de santé, jusqu'à concurrence de 700 CHF par an. La quote-part s'applique à toutes les prestations (y compris les médicaments) couvertes par l'assurance maladie de base et dépassant la franchise annuelle.* |
| *Franchise annuelle* | *La franchise représente un montant fixe jusqu'à concurrence duquel vous devez payer vous-même vos frais de santé par an. Ce montant fixe s'applique à tous les services (y compris les médicaments) couverts par l'assurance maladie de base.* |
| *Prime mensuelle* | *La prime mensuelle est le montant que vous payez chaque mois à la compagnie d'assurance maladie pour votre assurance de base. La prime mensuelle pour les adultes (âgés de 26 ans et plus) dépend du canton de résidence, de l'assureur, du modèle d'assurance et de la franchise. En 2020, les assureurs ont facturé une prime mensuelle moyenne d'environ 320 CHF pour l'assurance de base.* |

*Actuellement, l'assurance maladie rembourse des médicaments et services peu utiles aux patients ou qui sont très chers par rapport à leurs bienfaits. Une quote-part plus basse pour les médicaments et les services qui sont les plus bénéfiques, permettrait ainsi d’éviter des dépenses inutiles dans le système de santé et améliorerait la qualité des soins.*

*Nous allons maintenant vous présenter des scenarios montrant deux modèles d'assurance maladie (A et B). Veuillez choisir le modèle d'assurance qui vous convient le mieux. Veuillez simplement indiquer votre préférence spontanée ; il n'y a pas de bonnes ou de mauvaises réponses.*

## Deutche Version

### 3.1 Gruppe 1

*Bitte lesen Sie die folgenden Anweisungen genau, Sie sind wichtig für die weiteren Fragen.*

*In der Schweizer Gesundheitspolitik werden derzeit verschiedene Änderungen in der obligatorischen Grundversicherung diskutiert. Im Folgenden zeigen wir Ihnen einige hypothetische Versicherungsmodelle in der Grundversicherung und möchten gerne von Ihnen wissen, welches der gezeigten Modelle Sie bevorzugen würden.*

*In den Versicherungsmodellen geht es um folgende Merkmale: die monatliche Prämie, die jährliche Franchise und den jährlichen Selbstbehalt, sowie die Rückerstattung von Medikamenten. Anhand dieser Merkmale zeigen wir Ihnen jeweils zwei Versicherungsmodelle (A und B). Bitte wählen Sie das Versicherungsmodell aus, das am besten zu Ihnen passen würde. Bitte geben Sie einfach Ihre spontane Präferenz an; es gibt hier keine richtigen oder falschen Antworten.*

### 3.2 Gruppe 2

*Bitte lesen Sie die folgenden Anweisungen genau, Sie sind wichtig für die weiteren Fragen.*

*In der Schweizer Gesundheitspolitik werden derzeit verschiedene Änderungen in der obligatorischen Grundversicherung diskutiert. Im Folgenden zeigen wir Ihnen einige hypothetische Versicherungsmodelle in der Grundversicherung und möchten gerne von Ihnen wissen, welches der Modelle Sie bevorzugen würden.*

*In den Versicherungsmodellen geht es um folgende Merkmale:*

| *Rückerstattung von Medikamenten* | *Das Krankenversicherungsgesetz regelt, dass nur wirksame, zweckmässige und wirtschaftliche Medikamente in der Grundversicherung rückerstattet werden. Die gedeckten Medikamente werden in einer Liste geführt.* |
| --- | --- |
| *Jährlicher Selbstbehalt* | *Alle Versicherten sind verpflichtet, 10 Prozent Ihrer Gesundheitskosten bis maximal 700 CHF jährlich selbst zu bezahlen. Der Selbstbehalt gilt für alle Leistungen (inkl. Medikamente), die von der Grundversicherung gedeckt werden und über die jährliche Franchise hinausgehen.* |
| *Jährliche Franchise* | *Die Franchise stellt einen Fixbetrag dar, bis zu dem Sie Ihre eigenen Gesundheitskosten pro Jahr selbst bezahlen müssen. Dieser Fixbetrag gilt für alle Leistungen (inkl. Medikamente), die von der Grundversicherung gedeckt werden.* |
| *Monatliche Prämie* | *Die monatliche Prämie ist der Betrag, den Sie jeden Monat für Ihre Grundversicherung an die Krankenkasse bezahlen. Die monatliche Prämie für Erwachsene (ab 26 Jahren) hängt vom Wohnort, dem Versicherer, dem Versicherungsmodell und der Franchise ab. Im Jahr 2020 haben die Versicherer im Durchschnitt für die Grundversicherung eine Monatsprämie von etwa 320 CHF verlangt.* |

*Anhand dieser Merkmale zeigen wir Ihnen jeweils zwei Versicherungsmodelle (A und B). Bitte wählen Sie das Versicherungsmodell aus, das am besten zu Ihnen passen würde. Bitte geben Sie einfach Ihre spontane Präferenz an; es gibt hier keine richtigen oder falschen Antworten.*

### 3.3 Gruppe 3

*Bitte lesen Sie die folgenden Anweisungen genau, Sie sind wichtig für die weiteren Fragen.*

*In der Schweizer Gesundheitspolitik werden derzeit verschiedene Änderungen in der obligatorischen Grundversicherung diskutiert. Im Folgenden zeigen wir Ihnen einige hypothetische Versicherungsmodelle in der Grundversicherung und möchten gerne von Ihnen wissen, welches der Modelle Sie bevorzugen würden.*

*In den Versicherungsmodellen geht es um folgende Merkmale:*

| *Rückerstattung von Medikamenten* | *Das Krankenversicherungsgesetz regelt, dass nur wirksame, zweckmässige und wirtschaftliche Medikamente in der Grundversicherung rückerstattet werden. Die gedeckten Medikamente werden in einer Liste geführt.* |
| --- | --- |
| *Jährlicher Selbstbehalt* | *Alle Versicherten sind verpflichtet, 10 Prozent Ihrer Gesundheitskosten bis maximal 700 CHF jährlich selbst zu bezahlen. Der Selbstbehalt gilt für alle Leistungen (inkl. Medikamente), die von der Grundversicherung gedeckt werden und über die jährliche Franchise hinausgehen.* |
| *Jährliche Franchise* | *Die Franchise stellt einen Fixbetrag dar, bis zu dem Sie Ihre eigenen Gesundheitskosten pro Jahr selbst bezahlen müssen. Dieser Fixbetrag gilt für alle Leistungen (inkl. Medikamente), die von der Grundversicherung gedeckt werden.* |
| *Monatliche Prämie* | *Die monatliche Prämie ist der Betrag, den Sie jeden Monat für Ihre Grundversicherung an die Krankenkasse bezahlen. Die monatliche Prämie für Erwachsene (ab 26 Jahren) hängt vom Wohnort, dem Versicherer, dem Versicherungsmodell und der Franchise ab. Im Jahr 2020 haben die Versicherer im Durchschnitt für die Grundversicherung eine Monatsprämie von etwa 320 CHF verlangt.* |

*Aktuell werden in der Grundversicherung immer noch Medikamente und Leistungen erstattet, welche den Patientinnen und Patienten nur wenig nützen, oder im Vergleich zu deren Nutzen sehr teuer sind. Ein niedrigerer Selbstbehalt für Medikamente und Leistungen, die nachweislich am meisten Nutzen stiften, würde dabei helfen, unnötige Ausgaben im Gesundheitswesen zu vermeiden und die Versorgungsqualität zu erhöhen.*

*Wir zeigen Ihnen nun jeweils zwei Versicherungsmodelle (A und B). Bitte wählen Sie das Versicherungsmodell aus, das am besten zu Ihnen passen würde. Bitte geben Sie einfach Ihre spontane Präferenz an; es gibt hier keine richtigen oder falschen Antworten.*

## Versione italiana

### 4.1 Gruppo 1

*Si prega di leggere attentamente le seguenti istruzioni.*

*Nella politica sanitaria Svizzera sono attualmente in discussione diversi cambiamenti nell'assicurazione di base obbligatoria. Di seguito sono presentati alcuni modelli ipotetici per l’assicurazione di base. Vorremmo gentilmente sapere quale modello Lei preferisce.*

*I modelli assicurativi si concentrano sulle seguenti caratteristiche: il premio assicurativo mensile, il tasso di franchigia annuale e la quota parte annuale, così come il rimborso per i medicinali. In base a queste caratteristiche Le verranno mostrati due modelli assicurativi (A e B). Selezioni il modello assicurativo che preferisce. Si prega di fornire una risposta spontanea; non ci sono risposte giuste o sbagliate.*

### 4.2 Gruppo 2

*Si prega di leggere attentamente le seguenti istruzioni.*

*Nella politica sanitaria Svizzera sono attualmente in discussione diversi cambiamenti nell'assicurazione di base obbligatoria. Di seguito sono presentati alcuni modelli ipotetici per l’assicurazione di base. Vorremmo gentilmente sapere quale modello Lei preferisce.*

*I modelli assicurativi di base hanno le seguenti caratteristiche:*

| *Rimborso per i medicinali* | *La legge sull'assicurazione malattie stabilisce che solo i medicinali efficaci, appropriati e dal prezzo contenuto siano rimborsati dall'assicurazione malattie di base. Questi medicinali rimborsati sono raggruppati in una lista.* |
| --- | --- |
| *Quota parte annuale* | *Tutte le persone assicurate sono tenute a pagare di tasca propria il 10% delle spese sanitarie fino a un massimo di 700 CHF all'anno. La franchigia si applica a tutte le prestazioni (compresi i medicinali) che sono coperte dall'assicurazione di base e che superano la franchigia annuale.* |
| *Franchigia annuale* | *La franchigia rappresenta un importo fisso fino al quale si devono pagare le spese sanitarie di un anno. Questo importo fisso si applica a tutte le prestazioni (compresi i farmaci) che sono coperte dall'assicurazione di base.* |
| *Premio mensile* | *Il premio mensile è l'importo che paga ogni mese alla cassa malattia per la vostra assicurazione di base. Il premio mensile per gli adulti (dai 26 anni in su) dipende dal luogo di residenza, dall'assicuratore, dal modello di assicurazione e dal tasso di franchigia. Nel 2020, gli assicuratori hanno richiesto in media un premio mensile di circa 320 franchi per l'assicurazione di base.* |

*In base a queste caratteristiche Le verranno mostrati due modelli assicurativi (A e B). Selezioni il modello assicurativo che preferisce. Si prega di fornire una risposta spontanea; non ci sono risposte giuste o sbagliate.*

### 4.3 Gruppo 3

*Si prega di leggere attentamente le seguenti istruzioni.*

*Nella politica sanitaria Svizzera sono attualmente in discussione diversi cambiamenti nell'assicurazione di base obbligatoria. Di seguito sono presentati alcuni modelli ipotetici per l’assicurazione di base. Vorremmo gentilmente sapere quale modello Lei preferisce.*

*I modelli assicurativi di base hanno le seguenti caratteristiche:*

| *Rimborso per i medicinali* | *La legge sull'assicurazione malattie stabilisce che solo i medicinali efficaci, appropriati e dal prezzo contenuto siano rimborsati dall'assicurazione malattie di base. Questi medicinali rimborsati sono raggruppati in una lista.* |
| --- | --- |
| *Quota parte annuale* | *Tutte le persone assicurate sono tenute a pagare di tasca propria il 10% delle spese sanitarie fino a un massimo di 700 CHF all'anno. La franchigia si applica a tutte le prestazioni (compresi i medicinali) che sono coperte dall'assicurazione di base e che superano la franchigia annuale.* |
| *Franchigia annuale* | *La franchigia rappresenta un importo fisso fino al quale si devono pagare le spese sanitarie di un anno. Questo importo fisso si applica a tutte le prestazioni (compresi i farmaci) che sono coperte dall'assicurazione di base.* |
| *Premio mensile* | *Il premio mensile è l'importo che paga ogni mese alla cassa malattia per la vostra assicurazione di base. Il premio mensile per gli adulti (dai 26 anni in su) dipende dal luogo di residenza, dall'assicuratore, dal modello di assicurazione e dal tasso di franchigia. Nel 2020, gli assicuratori hanno richiesto in media un premio mensile di circa 320 franchi per l'assicurazione di base.* |

*Attualmente, l'assicurazione malattie di base rimborsa ancora farmaci e servizi che sono di scarsa utilità per i pazienti o sono molto costosi rispetto alla loro utilità. Una quota parte più bassa per le medicine e i servizi che si sono dimostrati più benefici aiuterebbe ad evitare spese mediche inutili e ad aumentare la qualità delle cure. In base a queste caratteristiche Le verranno mostrati due modelli assicurativi (A e B). Selezioni il modello assicurativo che preferisce. Si prega di fornire una risposta spontanea; non ci sono risposte giuste o sbagliate.*
