# Supplementary material for: Does targeted information impact consumers’ preferences for value-based health insurance? Evidence from a survey experiment
Source: Health Econ Rev. 2024 Nov 18;14:94. doi: 10.1186/s13561-024-00573-9 (PMC11571679; doi:10.1186/s13561-024-00573-9)
Supplement: Supplementary file 3 — Supplementary Material 3 [file 13561_2024_573_MOESM3_ESM.docx]

# Appendix 3


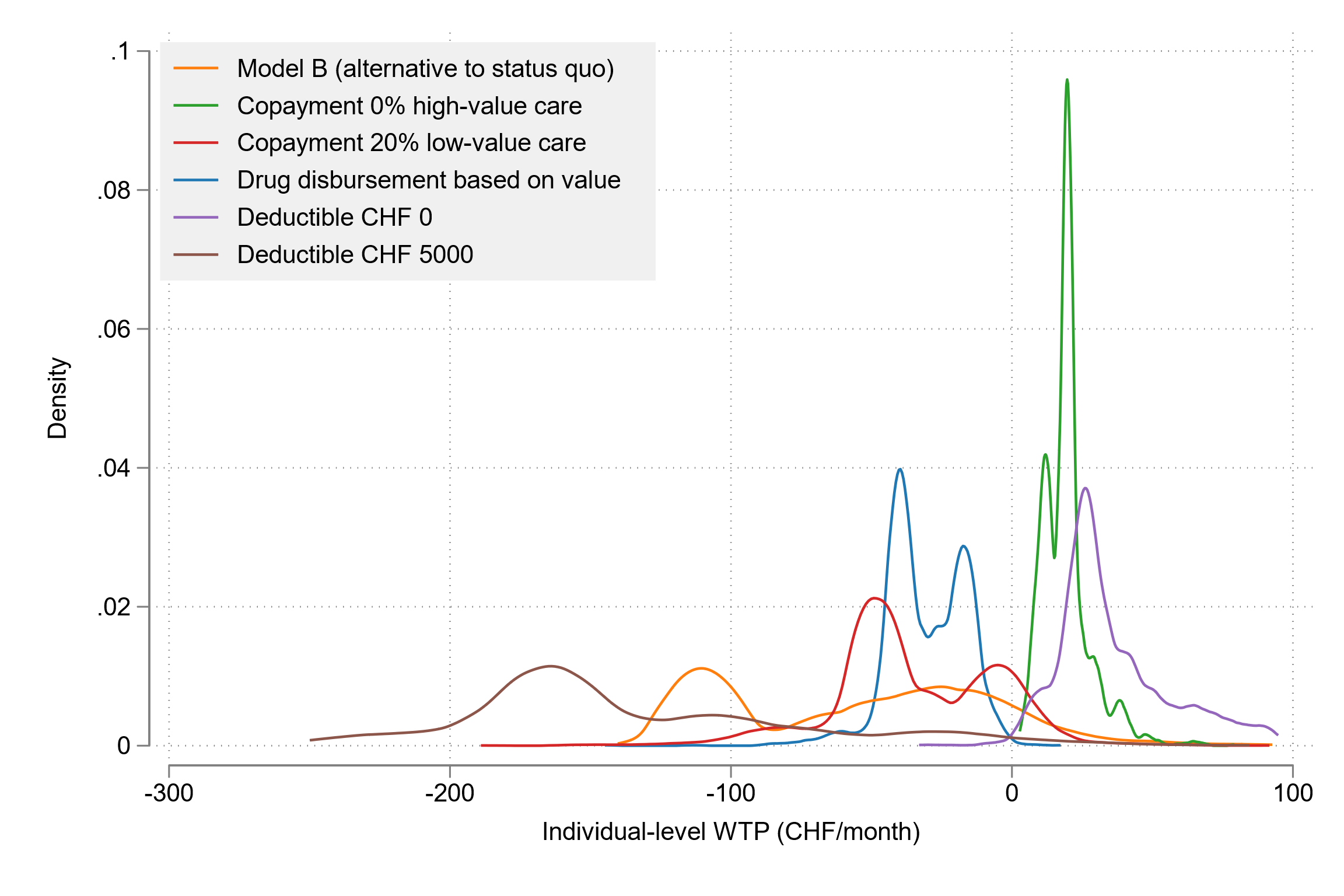
Control group


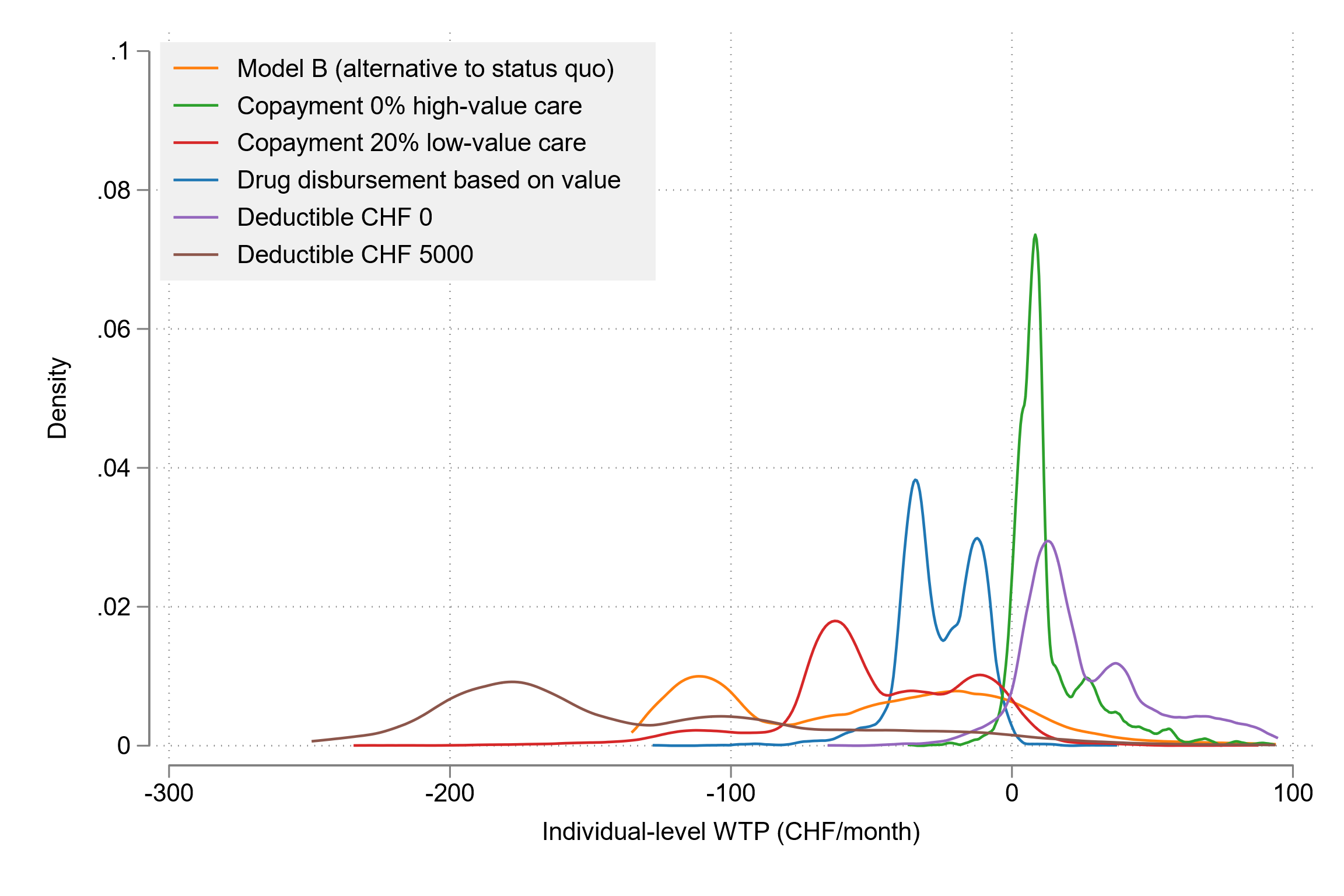

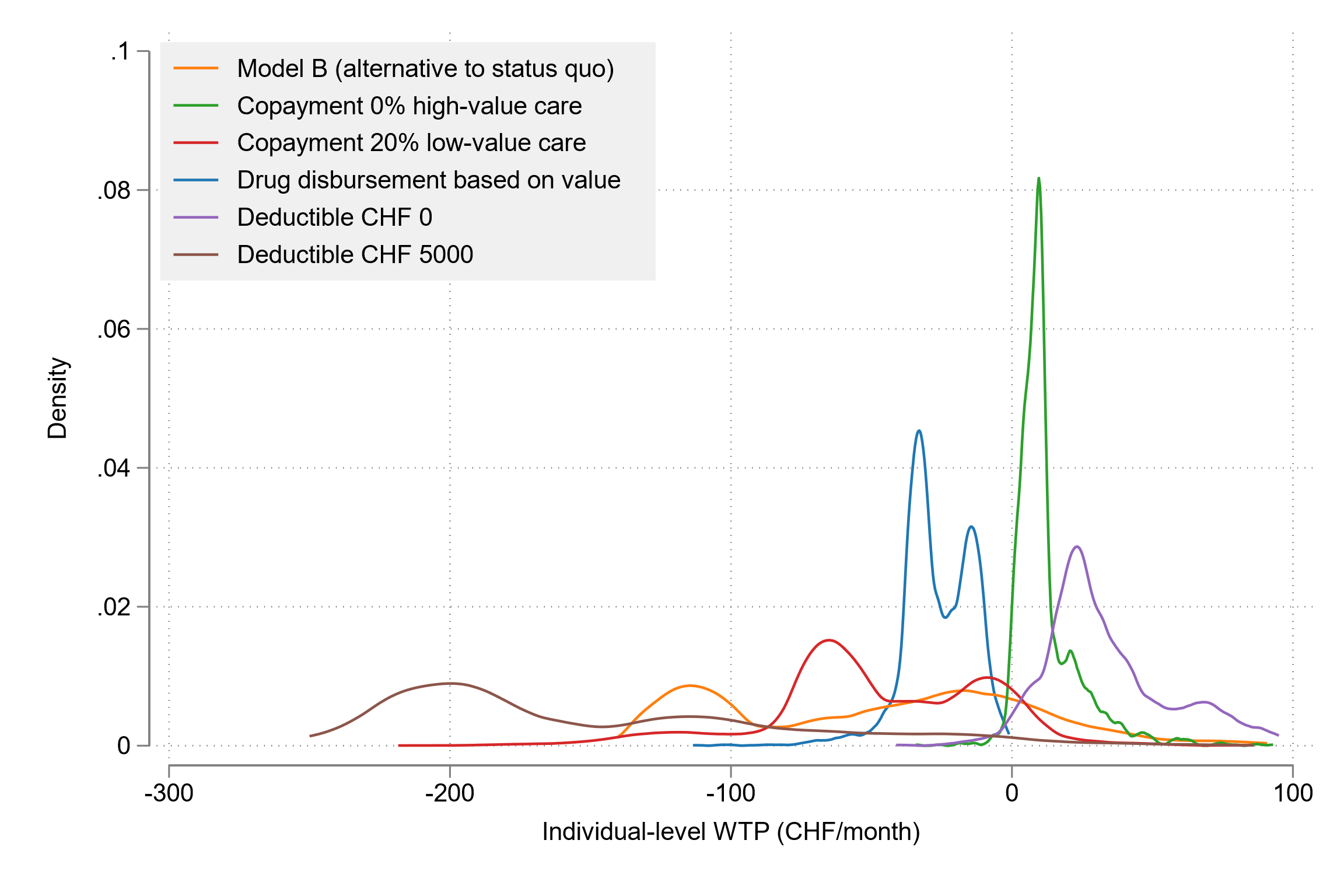
 Treatment group 1 Treatment group 2

Notes: WTP = willingness-to-pay; CHF = Swiss francs.

Figure A1: Individual-level willingness-to-pay distribution for the control and two treatment groups.
